# Fibulin-5 inhibits Wnt/β-catenin signaling in lung cancer

**DOI:** 10.18632/oncotarget.3609

**Published:** 2015-04-02

**Authors:** Xiaojun Chen, Xiaomeng Song, Wen Yue, Dongshi Chen, Jian Yu, Zhi Yao, Lin Zhang

**Affiliations:** ^1^ Department of Immunology, Tianjin Key Laboratory of Cellular and Molecular Immunology, Key Laboratory of Immuno Microenvironment and Disease of the Educational Ministry, Tianjin Medical University, Tianjin, P.R. China; ^2^ Department of Pharmacology and Chemical Biology, University of Pittsburgh Cancer Institute, University of Pittsburgh School of Medicine, Pittsburgh, PA, USA; ^3^ Department of Pathology, University of Pittsburgh Cancer Institute, University of Pittsburgh School of Medicine, Pittsburgh, PA, USA

**Keywords:** fibulin-5, lung cancer, invasion, MMP-7, β-catenin

## Abstract

Metastatic lung cancer is incurable and a leading cause of cancer death in the United States. However, the molecular mechanism by which lung cancer cells invade other tissues has remained unclear. We previously identified fibulin-5, an extracellular matrix protein, as a frequently silenced gene in lung cancer and a suppressor of cell invasion. In this study, we found fibulin-5 functions by inhibiting the Wnt/β-catenin pathway. The Cancer Genome Atlas (TCGA) datasets show a strong association between loss of fibulin-5 expression and poor outcomes of lung cancer patients, and also activation of the Wnt target genes *MMP-7* and *c-Myc*. Fibulin-5 impedes Wnt/β-catenin signaling by inhibiting extracellular signal-regulated kinase (ERK) to activate glycogen synthase kinase-3 β (GSK3β), which downregulates β-catenin and prevents its nuclear accumulation, leading to suppression of *MMP-7* and *c-Myc* expression. These effects of fibulin-5 are mediated by its amino-terminal integrin-binding RGD motif. Fibulin-5 also blocks Wnt/β-catenin signaling *in vivo* in H460 metastasis and H1299 tumor models. Furthermore, knockdown of *β-catenin* suppresses metastasis of H460 tumors, while knockdown of *GSK3β* promotes metastasis of fibulin-5-expressing H1752 tumors. Together, our results suggest that fibulin-5 functions as a metastasis suppressor in lung cancer by modulating tumor microenvironment to suppress Wnt/β-catenin signaling.

## INTRODUCTION

Lung cancer, accounting for nearly 27% of cancer deaths, is the leading cause of cancer death in the United States [1]. The high mortality rate of lung cancer is largely due to spread of disease to other organs [2]. Despite recent advances in early detection and targeted therapy, metastatic lung cancer remains incurable and results in poor patient outcomes. Understanding the molecular mechanism of lung cancer invasion and metastasis is crucial for developing novel and more effective therapeutic approaches.

The fibulin family, including fibulin-1–7, is a group of widely expressed extracellular matrix (ECM) proteins involved in cell motility, cell adhesion, elastogenesis, as well as cell-to-cell and cell-to-matrix communication [3]. Aberrant expression of the fibulin family proteins has been described in a variety of tumors [4]. Depending on cell types and cellular contexts, fibulin family proteins can either suppress or promote tumor growth [5, 6]. Fibulin-5, a 66-KD secreted glycoprotein, is localized in elastic fibers and essential for proper elastic fiber assembly and vasculogenesis [7]. It has a unique feature of containing an Arg-Gly-Asp (RGD) motif, which can bind to cell-surface receptors known as integrins [8]. Integrins not only mediate cell adhesion to the ECM, but also regulate intracellular signaling through kinases such as extracellular signal-regulated kinase (ERK) and focal adhesion kinase (FAK) [9]. We previously identified *fibulin-5* and *fibulin-3* as two genes that are most frequently silenced by promoter hypermethylation in non-small cell lung cancer (NSCLC) [10, 11]. Fibulin-5 and fibulin-3 suppress lung cancer invasion and metastasis by inhibiting the expression of matrix metalloproteinase 7 (MMP-7) [11, 12], which promotes tumor metastasis by degrading the basement membrane that serves as a barrier to surrounding tissues [13]. However, it is unclear how fibulin-5 inhibits MMP-7 expression and lung cancer cell invasion.

The Wnt/β-catenin pathway plays a critical role in tumor initiation, progression and metastasis [14]. The central player β-catenin is an oncoprotein normally localized in the cytoplasm, where it forms a complex with several proteins, including the APC tumor suppressor and glycogen synthase kinase 3β (GSK3β) [15]. This complex promotes β-catenin phosphorylation by GSK3β and its subsequent poly-ubiquitination and proteasomal degradation [15]. Oncogenic mutations, such as inactivating *APC* mutations in colon cancer, result in the stabilization and translocation of β-catenin to the nucleus, where it binds to the T-cell factor (TCF) family of transcription factors to induce the expression of downstream target genes, such as *c-Myc*, *CCND1*, and *MMP-7*. The Wnt/β-catenin pathway is frequently activated in lung cancer [16, 17], and contributes to lung cancer cell proliferation, survival and metastasis [17–20]. However, it is not fully understood how this pathway is aberrantly activated in lung cancer.

In this study, we found that loss of fibulin-5 expression in lung cancer is strongly associated with accumulation of β-catenin and activation of *MMP-7* and *c-Myc*. Fibulin-5, via its integrin-binding RGD motif, inhibits ERK to activate GSK3β and suppress β-catenin expression. Our results indicate that fibulin-5 functions as a metastasis suppressor in lung cancer by inhibiting Wnt/β-catenin signaling, and provide new insights on lung cancer invasion and metastasis.

## RESULTS

### Loss of fibulin-5 expression is correlated with poor outcomes of lung cancer patients

**Figure 1:**
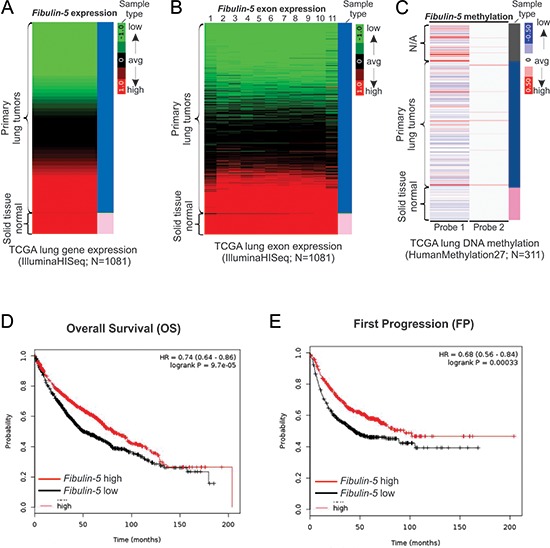
*Fibulin-5* downregulation is correlated with poor prognosis of lung cancer **A.** Heatmap of *fibulin-5* mRNA expression in the TCGA lung cancer (LUNG) RNAseq (IlluminaHiSeq; *N* = 1081) dataset. **B.** Heatmap of *fibulin-5* exon expression in the TCGA lung cancer (LUNG) RNAseq (IlluminaHiSeq; *N* = 1081) dataset. **C.** Heatmap of *fibulin-5* methylation in the TCGA lung cancer (LUNG) HumanMethylation27 (Illumina 27K platform; *N* = 311) dataset. **D.** Kaplan-Meier curves for comparing overall survival (OS) of patients with lung tumors expressing high and low expression levels of *fibulin-5*. **E.** Kaplan-Meier curves for comparing first progression (FP) of patients with lung tumors expressing high and low levels of *fibulin-5*.

*Fibulin-5* is known to be epigenetically silenced in lung cancer by promoter hypermethylation [11]. To further investigate its role in lung cancer, we analyzed the expression of *fibulin-5* in large datasets from The Cancer Genome Atlas (TCGA) databases. TCGA RNA Seq data showed marked downregulation of *fibulin-5* in over 50% of 1081 lung tumors analyzed, compared to normal control tissues, which all express high levels of *fibulin-5* (Fig. 1A). Expression of all 11 exons of *fibulin-5* is concomitantly reduced in lung tumors (Fig. 1B), suggesting altered regulation at its promoter. Consistent with our previous finding [11], hypermethylation in the genomic region of *fibulin-5* was detected by a probe in a substantial fraction of lung tumors (Fig. 1C). Importantly, loss of *fibulin-5* expression was found to be significantly associated with poor outcomes of lung cancer patients, including decreased overall survival (HR = 0.74; *P* = 9.7 × 10^−5^) (Fig. 1D), earlier first progression (HR = 0.68; *P* = 3.3 × 10^−4^) (Fig. 1E), and lower post-progression survival (HR = 0.75; *P* = 0.042) (Figure S1). These results, along with the association of fibulin-5 silencing and advanced tumors [11], strongly suggest that loss of *fibulin-5* expression plays an important role in lung cancer progression.

### *Fibulin-5* silencing is associated with elevated Wnt target genes and β-catenin in lung cancer

**Figure 2:**
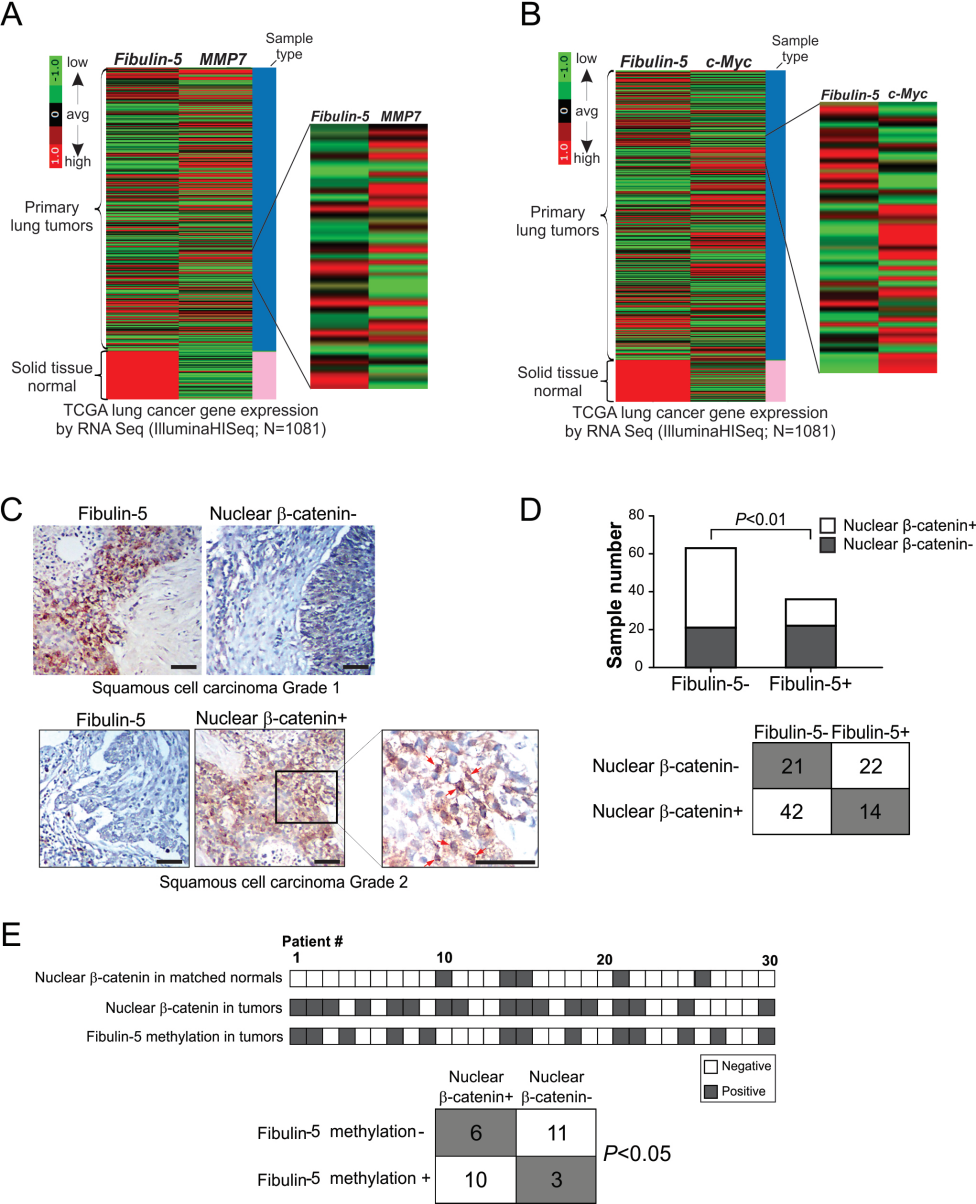
*Fibulin-5* silencing is correlated with activation of the Wnt/β-catenin pathway in lung cancer **A.** Heatmap for comparing *fibulin-5* and *MMP-7* mRNA expression in the TCGA lung cancer (LUNG) RNAseq (IlluminaHiSeq; *N* = 1081) dataset. **B.** Heatmap for comparing *fibulin-5* and *c-Myc* mRNA expression in the TCGA lung cancer (LUNG) RNAseq (IlluminaHiSeq; *N* = 1081) dataset. **C.** Analysis of fibulin-5 and β-catenin expression in NSCLC by immunostaining. Pictures show an example tumor that is positive for fibulin-5 but negative for nuclear β-catenin staining, and an example tumor that is negative for fibulin-5 but positive for nuclear β-catenin staining. Arrows in the enlarged field indicate example cells with nuclear β-catenin staining. Scale bar: 50 μm. **D.** Summary of fibulin-5 and nuclear β-catenin staining results in 99 NSCLC samples. The inverse correlation between fibulin-5 expression and nuclear β-catenin expression was significant (*P* = 0.0073, two-tailed χ2 test). **E.** *Top*, summary of nuclear β-catenin expression and *fibulin-5* promoter methylation in an independent set of 30 pairs of lung tumors and matched pathologically normal lung tissues. *Bottom*, correlation of nuclear β-catenin expression and *fibulin-5* promoter methylation in lung tumors (*P* = 0.0235, two-tailed χ2 test).

To understand how fibulin-5 suppresses lung cancer progression, we compared the expression of *fibulin-5* with other genes implicated in lung cancer progression. TCGA data showed an obvious inverse correlation between *fibulin-5* and *MMP-7* expression (Fig. 2A). A striking correlation between loss of fibulin-5 expression and induction of *c-Myc* was also noted; with most of the 1081 tumors analyzed expressing either low levels of *fibulin-5* but high levels of *c-Myc*, or high levels of *fibulin-5* but low levels of *c-Myc* (Fig. 2B).

Since both *MMP-7* and *c-Myc* are downstream effectors of the Wnt/β-catenin pathway [21], we hypothesized that the function of fibulin-5 in lung cancer is mediated through Wnt/β-catenin signaling. Upon analyzing an NSCLC tissue microarray by immunostaining (Fig. 2C and Table S1), we detected a statistically significant (*P* = 0.0073) association between loss of fibulin-5 expression and accumulation of nuclear β-catenin. While 64.6% (64/99) of tumors expressed either fibulin-5 or nuclear β-catenin, only 14.1% (14/99) were positive for both (Fig. 2D). Further analysis of an independent set of 30 matched tumor and normal pairs showed a significant (*P* = 0.0235) association of *fibulin-5* promoter methylation and β-catenin expression, with 76.9% (10/13) of tumors having *fibulin-5* promoter methylation and nuclear β-catenin staining, compared to 35.3% (6/17) lacking *fibulin-5* promoter methylation but expressing nuclear β-catenin (Fig. 2E). These results suggest that loss of fibulin-5 expression contributes to activation of the Wnt pathway components, including β-catenin, c-Myc, and MMP-7, in lung cancer.

### Fibulin-5 suppresses β-catenin nuclear localization and TCF4 activity in lung cancer cells

The functional role of fibulin-5 in suppression of lung cancer was investigated. Transfection of *fibulin-5* into fibulin-5-negative NSCLC cells, including A549, H1299 and H460 cells, inhibited colonogenic formation (Fig. S2A). Stable expression of fibulin-5 in A549 and H1299 cells suppressed cell invasion [11], as well as cell proliferation determined by MTT assay (Fig. S2, B and C). Nonetheless, fibulin-5 expression did not induce substantial apoptosis in lung cancer cells (data not shown).

**Figure 3:**
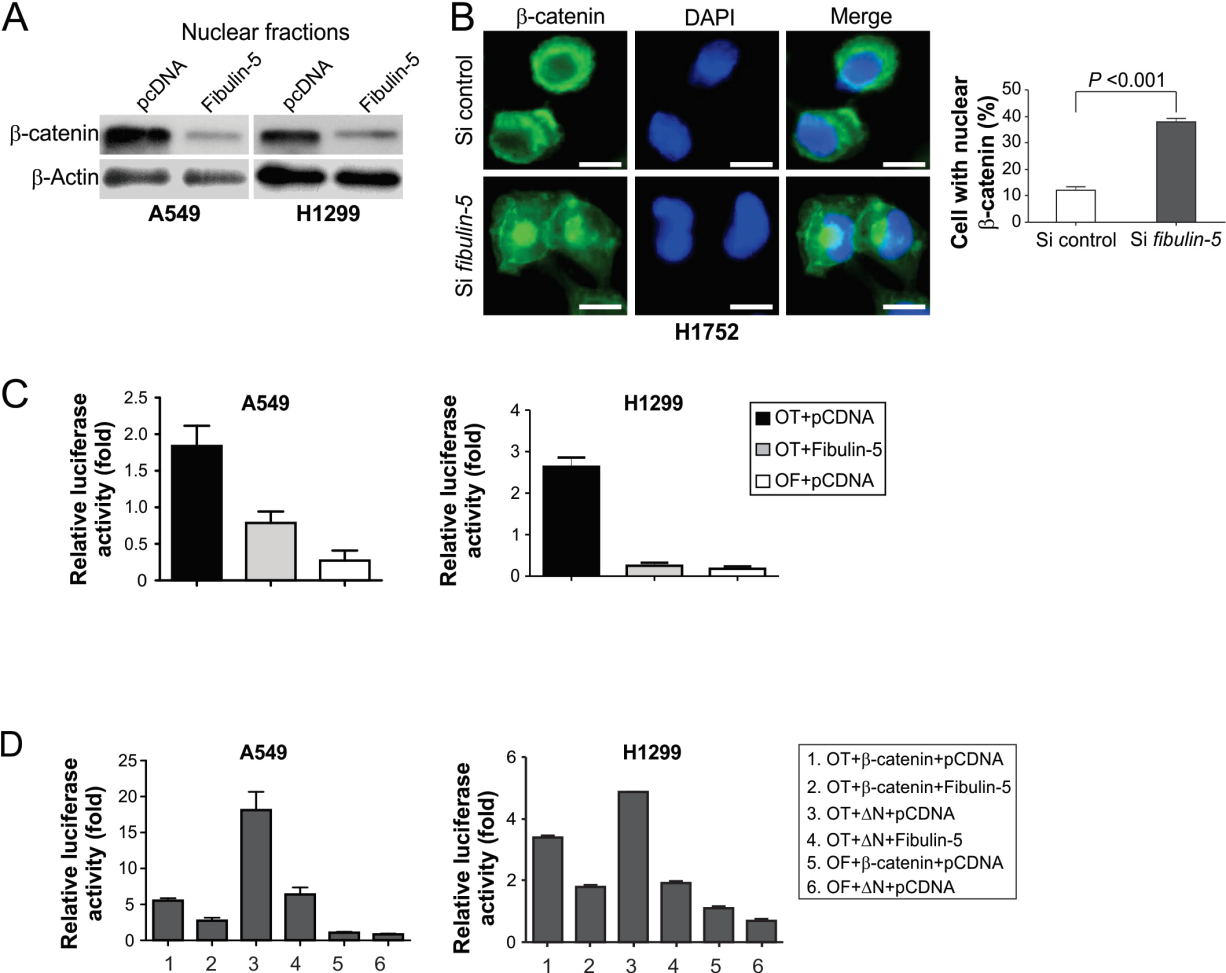
Fibulin-5 inhibits β-catenin nuclear translocation and TCF-4 activity **A.** Western blotting of β-catenin in nuclear fractions isolated from H1299 and A549 cells at 24 hr after transfection with *fibulin-5* or the control empty vector. **B.** H1752 cells transfected with *fibulin-5* or control siRNA were analyzed for β-catenin by immunostaining 48 hr after transfection. *Left*, representative pictures of β-catenin immunostaining. Scale bar, 5 μm. *Right*, quantification of cells with nuclear β-catenin (*P* = 0.0002, Student's *t* test). **C.** A549 and H1299 cells were transfected with *fibulin-5* along with the TCF-4 reporter pTOPFlash (OT) or the control inactive reporter pFOPFlash (OF). Normalized luciferase activities were determined 24 hr after transfection. The activity of the OF was defined as 1.0. **D.** The effects of fibulin-5 on TCF-4 reporter activities induced by WT or mutant β-catenin (ΔN). ΔN: the mutant β-catenin with amino-terminal 45 amino acids deleted. Reporter assays were performed as in (C).

We determined whether the effects of fibulin-5 on lung cancer invasion and MMP-7 expression are mediated by Wnt/β-catenin signaling, which is known to regulate MMP-7 expression in colorectal cancer cells [21]. Fibulin-5 expression substantially decreased the level of nuclear β-catenin in A549 and H1299 cells (Fig. 3A), and in SW480 colorectal cancer cells, which express abundant nuclear β-catenin (Fig. S3). Knockdown of *fibulin-5* by siRNA in H1752 lung cancer cells, which express endogenous fibulin-5, was sufficient to induce the expression of nuclear β-catenin (Fig. 3B), and also promote cell invasion [11]. Furthermore, transfection of *fibulin-5* into A549 and H1299 cells, but not the control vector, inhibited the activity of the TOPFlash (OT) TCF-4 reporter, without affecting the control FOPFlash (OF) reporter (Fig. 3C). Fibulin-5 also inhibited transactivation of the TOPFlash reporter by wild-type (WT) β-catenin, and that by a tumor-derived mutant β-catenin lacking the amino-terminal phosphorylation sites (ΔN) required for its degradation (Fig. 3D). These results suggest that fibulin-5 inhibits the Wnt pathway by preventing β-catenin accumulation and nuclear translocation.

### Fibulin-5 inhibits the Wnt/β-catenin pathway to suppress lung cancer cell invasion and MMP-7 expression

**Figure 4:**
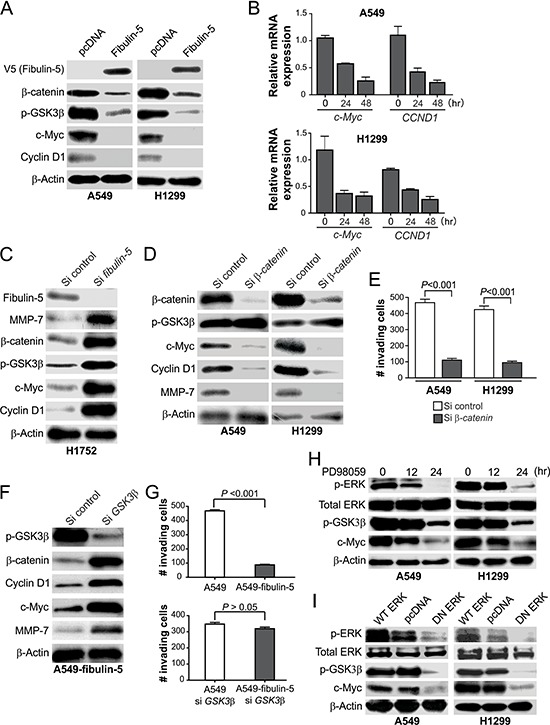
Fibulin-5 suppresses Wnt target gene expression by inhibiting ERK and GSK3β phosphorylation **A.** Western blot analysis of the indicated proteins in H1299 and A549 cells 24 hr after transfection with *fibulin-5* or the control empty vector. **B.** Real-time RT-PCR analysis of *c-Myc* and *CCND1* mRNA expression in H1299 and A549 cells at indicated time points after *fibulin-*5 transfection. *Glyceraldehyde-3-phosphate dehydrogenase* (*GAPDH*), a house-keeping gene, was used as an internal control. The results were normalized to the cells without *fibulin-5* transfection (0 hr), which were defined as 1.0. **C.** Western blot analysis of the indicated proteins in H1752 cells at 48 hr after transfection with control or *fibulin-5* siRNA. **D.** Western blot analysis of the indicated proteins in A549 and H1299 cells 36 hr after transfection with control or *β-catenin* siRNA. **E.** Matrigel invasion analysis of A549 and H1299 cells transfected with control or *β-catenin* siRNA as in (D). The results are the average of three independent experiments (*P* = 0.0001 for both si control and si *β-catenin*, Students' *t* test). **F.** Western blot analysis of the indicated proteins in stable fibulin-5-expressing A549 cells (A549-fibulin-5) 24 hr after transfection with control or *GSK3β* siRNA. **G.** Matrigel invasion analysis of the parental and stable fibulin-5-expressing A549 cells with or without *GSK3β* knockdown as in (F) (si control, *P* = 0.0001; si *GSK3β, P* = *0.1264*, Student's *t* test). **H.** Western blot analysis of the indicated proteins in A549 cells and H1299 cells treated with 50 μM of the ERK inhibitor PD98059 for the indicated time. **I.** Western blot analysis of the indicated proteins in A549 cells and H1299 cells 24 hr after transfection with wild-type (WT) *ERK*, dominant negative (DN) *ERK*, or the control empty vector.

We further examined whether and how fibulin-5 regulates the Wnt/β-catenin pathway to suppress lung cancer invasion. Fibulin-5 transfection markedly decreased the protein and mRNA expression of *c-Myc* and *CCND1*, two major TCF-4 downstream targets [22, 23] (Fig. 4A and 4B). Conversely, knockdown of *fibulin-5* by siRNA in H1752 cells stimulated the expression of β-catenin, c-Myc, cyclin D1 and MMP-7 (Fig. 4C). Similar to *fibulin-5* transfection (Fig. 4A) [11], knockdown of *β-catenin* by siRNA in A549 and H1299 cells suppressed the expression of c-Myc, cyclin D1 and MMP-7 (Fig. 4D), as well as cell invasion determined by the Matrigel invasion assay (Fig. 4E and S4A).

β-catenin level is primarily regulated by ubiquitin/proteasome-mediated protein degradation, following its phosphorylation by GSK3β [15], whose kinase activity is governed by the inhibitory Ser9 phosphorylation [24]. Ser9 phosphorylation of GSK3β was markedly reduced following fibulin-5 transfection in A549 and H1299 cells (Fig. 4A), while increased in fibulin-5-depleted H1752 cells (Fig. 4C), but barely changed upon β-catenin depletion (Fig. 4D). Knockdown of *GSK3β* by siRNA in the stable fibulin-5-expressing A549 cells restored expression of β-catenin, c-Myc, cyclin D1 and MMP-7 (Fig. 4F), and invasive capacity (Fig. 4G and S4B).

The effect of fibulin-5 on invasion is mediated by inhibition of ERK kinase [11], which promotes Ser9 phosphorylation of GSK3β [24]. Similar to fibulin-5 transfection, the ERK inhibitor PD98059 suppressed GSK3β Ser9 phosphorylation and c-Myc expression in both A549 and H1299 cells (Fig. 4H). Transfecting A549 and H1299 cells with dominant negative (DN) ERK, but not wild-type ERK, also inhibited GSK3β phosphorylation and c-Myc expression (Fig. 4I). Together, these results indicate that fibulin-5 suppresses MMP-7 expression and lung cancer invasion through ERK inhibition and GSK3β activation, which in turn prevents β-catenin accumulation and nuclear translocation.

### Fibulin-5 downregulates MMP-7 through its integrin-binding RGD motif

**Figure 5:**
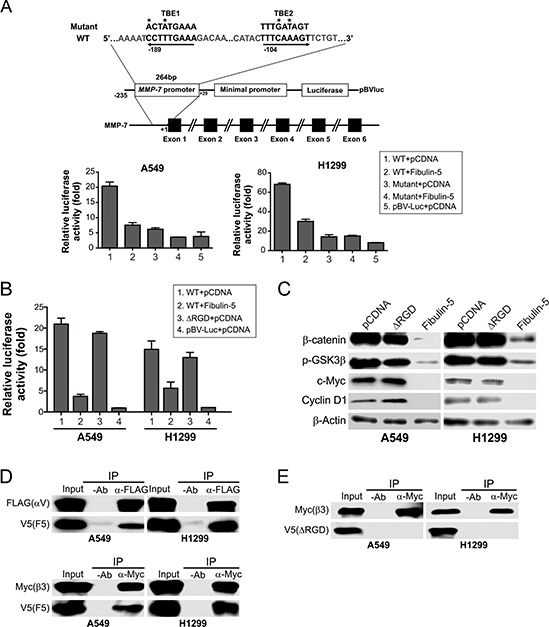
Fibulin-5 suppresses MMP-7 expression through its integrin-binding RGD motif **A.** *Upper*, schematic representation of *MMP-7* promoter region containing two TCF4 binding elements (TBEs). Asterisks indicate the mutated nucleotides. *Lower*, A549 and H1299 cells were transfected with the control (pBV-Luc) or *MMP-7* reporter, along with *fibulin-5* or the empty pCDNA vector. Luciferase activities were measured 24 hr after transfection and normalized to that of the β-galactosidase reporter. **B.** A549 and H1299 cells were transfected with the control (pBV-Luc) or *MMP-7* reporter, along with WT *fibulin-5*, *fibulin-5* with the RGD domain deletion (ΔRGD), or the empty pCDNA vector. Luciferase activities were measured as in (A). **C.** Western blotting of the indicated proteins in A549 and H1299 cells 24 hr after transfection with WT or ΔRGD *fibulin-5*. **D.** A549 and H1299 cells were co-transfected with V5-tagged fibulin-5, along with Flag-tagged αv or Myc-tagged β3. IP was performed with anti-Flag or anti-Myc antibody, followed by western blotting of the indicated proteins. **E.** IP was performed on A549 and H1299 cells co-transfected with V5-tagged ΔRGD along with Flag-tagged αv or Myc-tagged β3.

There are at least two TCF-4 binding elements (TBEs) in the promoter region of *MMP-7* [12]. *Fibulin-5* transfection markedly inhibited activity of an *MMP-7* reporter construct containing these TBEs in A549 and H1299 cells, but not that of a reporter containing point mutations in both TBEs (Fig. 5A), suggesting the inhibitory effect of fibulin-5 on *MMP-7* is mediated at the transcriptional level through the TBEs.

Fibulin-5 is distinguished from other fibulin family members in its unique RGD motif. Inhibition of the Wnt pathway by fibulin-5 requires the RGD motif, as the mutant fibulin-5 lacking the RGD motif (ΔRGD) was unable to suppress the *MMP-7* reporter (Fig. 5B), GSK3β Ser9 phosphorylation, or c-Myc and cyclin D1 expression in A549 and H1299 cells (Fig. 5C). We then used IP to probe potential interactions between fibulin-5 and integrins, which can bind to RGD-containing proteins [9]. While WT fibulin-5 could bind to the integrin subunits αv and β3 in A549 and H1299 cells (Fig. 5D), ΔRGD could not interact with either subunit (Fig. 5E). Therefore, the inhibitory effect of fibulin-5 on the Wnt pathway in lung cancer is mediated by the integrin-binding RGD motif.

### Fibulin-5 suppresses tumor growth and metastasis and Wnt/β-catenin signaling *in vivo*

**Figure 6:**
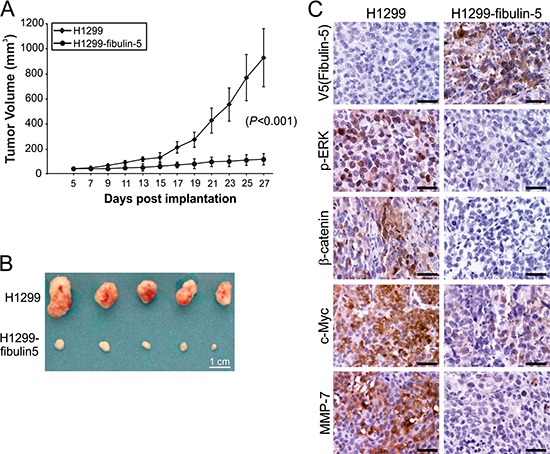
Fibulin-5 inhibits lung tumor progression and the Wnt pathway in mice **A.** Parental and fibulin-5-expressing H1299 cells were injected subcutaneously into BALB/c nude mice to establish xenograft tumors. Tumor volumes at indicated time points after inoculation were calculated and plotted (*n* = 5 in each group). The difference between parental and fibulin-5-expressing tumors was statistically significant (*P* = 0.0007, Fisher's exact test). **B.** Representative pictures of tumors at the end of experiments. **C.** Immunostaining analysis of V5 (fibulin-5), p-ERK, β-catenin, c-Myc, and MMP-7 in tumor tissues from (B). Scale bar, 20 μm.

**Figure 7:**
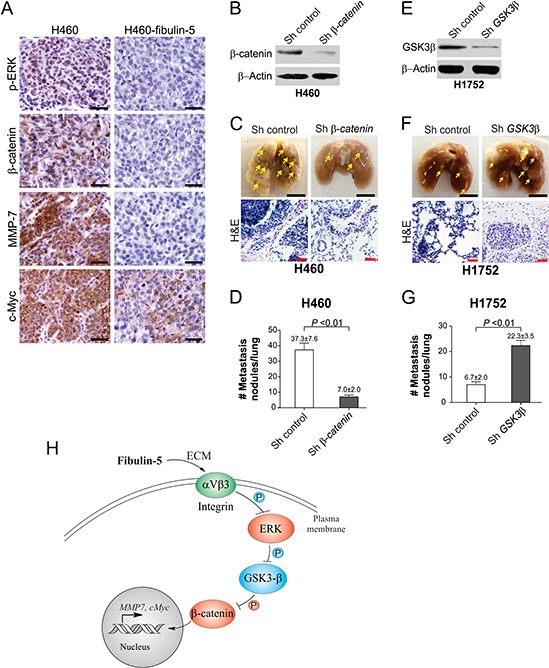
The Wnt pathway can be inhibited by fibulin-5 in metastatic tumors in mice and is critical for tumor metastasis **A.** Representative immunostaining pictures of p-ERK, β-catenin, c-Myc, and MMP-7 in the parental and fibulin-5-expressing H460 xenograft tumors. Scale bar, 20 μm. **B.** Western blot analysis of β-catenin in H460 cells expressing *β-catenin* or control shRNA. **C.** H460 cells expressing *β-catenin* or control shRNA were injected (10^6^ cells/injection) i.v. by tail vein into BALB/c nude mice. Representative pictures of fixed lungs at 6 weeks after injection were shown (Scale bar, 0.5 cm), along with H&E staining pictures (Scale bar, 50 μm). **D.** Quantification of metastasis nodules in H460 tumors in (C). The difference between the two groups was statistically significant (*P* = 0.0026, Student's *t* test). **E.** Western blot analysis of GSK3β in H1752 cells expressing *GSK3β* or control shRNA. **F.** H1752 cells expressing *β-catenin* or control shRNA were injected (10^6^ cells/injection) i.v. by tail vein into BALB/c nude mice. Representative pictures of fixed lungs at 6 weeks after injection were shown (Scale bar, 0.5 cm), along with H&E staining pictures (Scale bar, 50 μm). **G.** Quantification of metastasis nodules in H1752 tumors in (F). The difference between the two groups was statistically significant (*P* = 0.0028, Student's *t* test). **H.** A model of fibulin-5-mediated suppression of lung cancer cell invasion and proliferation.

Xenograft tumor models were used to further determine the role of fibulin-5 in suppressing lung tumor growth *in vivo*. Fibulin-5 expression significantly (*P* = 0.0007) suppressed the growth of H1299 xenograft tumors in nude mice (Fig. 6A and 6B). In line with reduced tumor growth, expression of p-ERK, nuclear β-catenin, c-Myc and MMP-7 was found to be substantially decreased in fibulin-5-expressing H1299 tumors, compared to the parental H1299 tumors lacking fibulin-5 expression (Fig. 6C). Because A549 and H1299 cells do not form lung metastasis, we used H460 and H1752 cells to analyze the effect of fibulin-5 on tumor metastasis. We found that lung metastasis, established from fibulin-5-expressing H460 cells by intravenous injection [11], also expressed much lower levels of p-ERK, nuclear β-catenin, c-Myc and MMP-7, relative to the parental H460 tumors (Fig. 7A). Knockdown of *β-catenin* in H460 cells by using lenti-viral shRNA significantly suppressed lung metastasis of H460 tumors (Fig. 7B–D). Furthermore, knockdown of *GSK3β* in H1752 cells by shRNA was sufficient to promote lung metastasis of H1752 tumors (Fig. 7E–G). These findings indicate that fibulin-5 functions as a suppressor of lung tumor growth and metastasis *in vivo* by inhibiting the Wnt/β-catenin pathway.

## DISCUSSION

Fibulin-5 is frequently downregulated in more than 50% of lung cancer at least in part due to promoter hypermethylation (Fig. 1A–C) [11]. Loss of fibulin-5 expression is associated with poor survival of lung cancer patients and disease progression (Fig. 1D and S1). The anti-tumor effect of fibulin-5 is mediated through tumor microenvironment and suppression of MMP-7, which is overexpressed in NSCLC and associated with poor prognosis [29, 30]. In addition to MMP-7, fibulin-5 can also regulate other metalloproteinases. It inhibits MMP-2, MMP-3, TIMP-1, and TIMP-3 in preventing tumor angiogenesis [31], and MMP-9 through an autocrine loop in blocking lung and liver metastasis mediated by stromal fibroblasts [32]. However, several studies showed tumor-promoting activity of fibulin-5 [33, 34], suggesting cell type- and context-dependent functions of fibulin-5 in cancer. The prominent role of fibulin-5 in lung cancer is in line with its function in pulmonary physiology and pathology. For example, altered fibulin-5 expression has been linked to lung injury and pulmonary hypertension [35, 36]. Fibulin-5-deficient mice primarily exhibited pulmonary phenotypes such as lung emphysemas, a risk factor of lung cancer [8, 37].

Wnt/β-catenin signaling has recently emerged as a critical player in lung cancer [38]. Some lung cancer cells express high levels of nuclear β-catenin, resulting in induction of Wnt target genes and enhanced cell proliferation [16, 17]. Our results indicate that fibulin-5 functions as an inhibitor of Wnt/β-catenin signaling in lung cancer, as indicated by the strong association of loss of fibulin-5 expression and accumulation of β-catenin, MMP-7 and c-Myc in lung tumors (Fig. 2), and suppression of the Wnt pathway components by fibulin-5 in NSCLC cells (Figs. 3–5). Fibulin-5 does not seem to be directly involved in β-catenin degradation, as it inhibits both WT and mutant β-catenin lacking the phosphorylation sites required for its degradation (Fig. 3D). It may facilitate retaining of β-catenin in the cytoplasm, where it binds to several other proteins that promote its subsequent turnover. In addition to fibulin-5 silencing, several other aberrations in lung cancer can lead to activation of the Wnt pathway, such as overexpression of Wnt ligands [20], downregulation of Wnt antagonists such as dickkopf 3 [10], and loss of fibulin-3 expression [12]. Since lung cancer cells generally lack *APC* and *β-catenin* mutations that are frequently found in colon tumors, they probably have to rely on multiple changes in the Wnt pathway regulators, which may complement each other in sustaining high levels of Wnt signaling.

Although c-Myc is frequently overexpressed, the amplification *c-Myc* genomic region was rare in NSCLC [39]. Our results suggest that fibulin-5 silencing contributes to c-Myc overexpression in lung cancer. Suppression of lung cancer metastasis by fibulin-5 may be mediated in part through c-Myc, which can promote lung cancer metastasis in mice [40]. Fibulin-5 may also be involved in regulating Wnt signaling in response to lung injury, in which Wnt signaling and MMP-7 were found to be concomitantly regulated [41].

Our results suggest that fibulin-5 antagonizes Wnt signaling by inhibiting ERK and indirectly activating GSK3β to restrain β-catenin in the cytoplasm, which prevents MMP-7 and c-Myc expression and lung cancer cell invasion and proliferation (Fig. 7H). The effect of fibulin-5 is likely to be mediated by its binding to cell-surface integrins, as it requires the RGD motif that binds to αvβ3 in lung cancer cells (Fig. 5B–E). Integrin-mediated signaling is known to be involved in cell migration and invasion, as well as activation of MMPs and TIMPs [42]. Fibulin-5 has been shown to bind to the integrins αvβ3, αvβ5, and α9β1 to promote endothelial cell adhesion [8]. It is possible that the effect of fibulin-5 in lung cancer involves integrins other than αvβ3, which will be investigated in our future studies. The RGD motif of fibulin-5 may also be useful for developing peptide mimetic or small molecule inhibitors for targeting metastatic lung cancer. Activation of the Wnt pathway due to fibulin-5 silencing may help drive epithelial-mesenchymal transition (EMT), a critical event in cancer metastasis, which is known to involve aberrant Wnt signaling, ERK signaling, and MMP activation [43]. Fibulin-5 was found to regulate EMT as a target of transforming growth factor β (TGFβ) [34]. These observations made by us and other groups will likely stimulate further interest in delineating how fibulin-5 silencing drives EMT-related events during lung cancer invasion and metastasis.

Collectively, our results demonstrate an important functional role of fibulin-5 in suppressing Wnt/β-catenin signaling and lung cancer invasion. Further investigation of the functional role of fibulin-5 may provide useful information for developing biological or pharmacological agents for targeting metastatic lung cancer.

## MATERIALS AND METHODS

### Bioinformatics analysis

*Fibulin-5*, *MMP-7* and *c-Myc* expression and *fibulin-5* DNA methylation in The Cancer Genome Atlas (TCGA) databases were analyzed by using The UCSC Cancer Genomics Browser (https://genome-cancer.soe.ucsc.edu/proj/site/hgHeatmap/) [25]. The TCGA lung cancer (LUNG) RNAseq (IlluminaHiSeq; *N* = 1081) and DNA methylation (HumanMethylation27; Illumina 27K platform; *N* = 311) datasets were used to compare *fibulin-5*, *MMP-7* and *c-Myc* expression and *fibulin-5* DNA methylation, because these datasets include results from control normal tissues. Heatmap mode was used to display the results. Kaplan-Meier curves were generated using the Kaplan-Meier plotter program (http://kmplot.com/analysis/) as described [26].

### Tissue samples and immunostaining

Tissue microarray slides containing 99 NSCLC samples were purchased from US Biomax (Rockville, MD). The information of these samples was summarized in Table S1. An independent set of frozen specimens with known *fibulin-5* promoter methylation status, including 30 randomly selected lung tumors and the matched histologically normal lung parenchyma samples, were previously described [11]. Fibulin-5 and β-catenin immunostaining was performed as described [10]. The staining distribution was scored based on the percentage of positive cells: 0, 0%; 1, 1–30%; 2, 31–60%; 3, 61–100%. The criteria used for scoring signal intensity were: 0, no signal; 1, weak; 2, moderate; and 3, marked. The staining was considered to be positive if the sum of distribution and intensity scores was > 2.

### Cell culture and transfection

Lung cancer cell lines were obtained from American Type Culture Collection (Manassas, VA). Cells were maintained at 37°C and 5% CO_2_, and cultured in RPMI 1640 medium (Mediatech, Herndon, VA) supplemented with 10% defined fetal bovine serum (HyClone, Logan, UT), 100 U/ml penicillin and 100 μg/ml streptomycin (Invitrogen, Carlsbad, CA). Transfection of plasmids and small interfering RNA (siRNA) was performed using Lipofectamine™ 2000 (Invitrogen) as described [27]. Expression vectors including wild-type ERK (plasmid #49328) and dominant negative (DN) ERK (K71R; plasmid #49329) were obtained from Addgene (Cambridge, MA). Fibulin-5 was knocked down by ON-TARGET plus siRNA J-017621-05 (ThermoFisher, Waltham, MA). *β-catenin* and *GSK3β* in A549 and H1299 cells were knocked down using pre-made siRNA (Santa Cruz Biotechnology, Santa Cruz, CA). *β-catenin* and *GSK3β* in H460 and H1752 cells were stably knocked down by transduction with pre-made lenti-viral short hairpin RNA (shRNA) vectors (Shanghai Genechem, Shanghai, China) as previously described [28]. The shRNA vectors included shRNA #18745 for *β-catenin* (target sequence: 5′-TTGGAATGAGACTGCTGAT-3′) and shRNA #9374 for *GSK3β* (target sequence: 5′-ACTGATTATACCTCTAGTA-3′). The infected cells were selected by puromycin at 5 μg/mL for H460 cells, and 3 μg/mL for H1752 cells. Cell viability was analyzed for cells seeded at 2 × 10^3^/well in 96-well plates using MTT assay kit (Promega, Madison, WI) according to the manufacturer's instructions.

### Western blots and immunoprecipitation

Collection of cell lysates and western blotting were done as previously described (24). The antibodies used for western blotting included those for p-GSK3β (Ser9), ERK, p-ERK (Thr202/Tyr204) (Cell Signaling Technology, Danvers, MA), cyclin D, c-Myc (9E10) (Santa Cruz Biotechnology), MMP7 (EMD Millipore, Billerica, MA), V5 (Invitrogen), β-actin (Sigma, St. Louis, MO), and β-catenin (BD Biosciences, San Jose, CA).

For immunoprecipitation (IP), cells were harvested by centrifugation and washed once with PBS on ice. Cells were lysed for 15 min with RIPA buffer (150 mM NaCl, 50 mM Tris, 1% NP-40, and 0.5% deoxycholate) containing 1:100 protease/phosphatase inhibitors (Sigma). After vortexing, the lysates were cleared by centrifugation, and then frozen at −80°C or used immediately for IP. For each IP reaction, lysate containing 500–600 mg proteins was used. Briefly, cell lysates were incubated with 2 mg of anti-FLAG (Sigma) or anti-Myc (9E10; Santa Cruz Biotechnology) at 4°C overnight, and then incubated with protein-G sepharose (Life technologies, Grand Island, NY) at room temperature for 2 hr with rocking. After centrifugation, the beads were washed once with 500 μL RIPA buffer containing protease/phosphatase inhibitors, 3 times with 1 mL of RIPA buffer, and then boiled in 2× Laemmli sample buffer. Whole cell lysates prepared before IP were used as a control for input. Samples were run on an SDS-PAGE pre-cast gels at 100 V for 1 hr (Bio-Rad, Hercules, CA), blotted onto Immobilon-P PVDF membranes (EMD Millipore), and then blocked with either 5% non-fat dry milk or 5% BSA diluted in wash buffer (0.1% tween-20 in TBS). After incubation with primary and secondary antibodies at 4°C, blots were developed using the ECL chemiluminescent detection kit (Amersham Biosciences, Buckinghamshire, UK).

### Reverse transcriptase (RT)-polymerase chain reaction (PCR)

Total RNA was isolated from lung cancer cells using the RNAgents Total RNA Isolation System (Promega). First-strand cDNA was synthesized from 10 μg of total RNA using Superscript II reverse transcriptase (Invitrogen). The PCR primers include those for *CCND1*, 5′-CCCTCGGTGTCCTACTTCAAA-3′/5′ CCAGGTTCCACTTGAGCTTGT-3′ and *c-Myc*, 5′-CCTCAACGTTAGCTTCACCAA-3′/5′-TTTGATGAA GGTCTCGTCGTC-3′. PCR reactions were performed on an MJ Mini Personal thermo cycler (Bio-Rad) with *glyceraldehyde-3-phosphate dehydrogenase* (*GAPDH*) as the internal control. The cycle conditions are available upon request.

### Matrigel invasion assay

Invasion assays were performed in triplicate in six-well trans-well units with 8-μm filters coated with Matrigel (BD Biosciences) at 1:6 dilution. Each well was loaded with ~2 × 10^6^ cells. After incubation for 36 hr, cells passing through the filters into bottom wells were fixed with formalin and stained with crystal violet (Sigma). Cell numbers in 10 randomly selected fields (×200) from each well were counted.

### Luciferase assay

WT and mutant *MMP-7* luciferase reporters constructed using the pBV-Luc vector were previously described [12]. For reporter assays, A549 and H1299 cells were co-transfected with fibulin-5 and the transfection control β-galactosidase reporter pCMVβ (Promega), along with the TCF-4 reporter plasmid pTOPFlash or the control inactive reporter pFOPFlash, and WT or mutant *MMP-7* reporter. In some experiments, cells were transfected with mutant fibulin-5 with deletion of its RGD motif (ΔRGD). After transfection, collection of cell lysates and measurement of luciferase activities were done as described [12]. All reporter experiments were performed in triplicate and repeated three times.

### Analysis of β-catenin nuclear localization

Nuclear β-catenin in H1299 and A549 cells was analyzed by western blotting of β-catenin in nuclear fractions isolated from transfected cells using the NE-PER nuclear/cytoplasmic extraction kit (ThermoFisher) according to the manufacturer's instructions. Nuclear β-catenin in SW480 colorectal cancer cells and H1752 lung cancer cells were analyzed using immunofluorescence as previously described [10]. Mounted slides were subjected to microscopic analysis under a Nikon fluorescence microscope (TS800) equipped with a SPOT camera and imaging software.

### Animal experiments

All animal experiments were approved by the Institutional Animal Care and Use Committees at the University of Pittsburgh and Tianjin Medical University. Female 5- to 6-week-old BALB/c nude mice (Charles River, Wilmington, MA or Military Academy of Medical Sciences Laboratory Animal Center, Beijing, China) were housed in a sterile environment with microisolator cages and allowed access to water and chow *ad libitum*. To analyze tumor growth, mice were injected subcutaneously in both flanks with parental and fibulin-5-expressing H1299 cells (5 × 10^6^ cells/injection). Tumor volumes were calculated according to the formula 0.5 × length × width^2^. To analyze tumor metastasis, stable H460 and H1752 cells expressing lenti-viral *β-catenin*, *GSK3β*, or control scrambled shRNA were injected intravenously (i.v.) by tail vein into BALB/c nude mice. For each injection, 1 × 10^6^ cells suspended in 200 μl PBS were used. Following sacrifice of mice at 6 weeks after injection, lung metastasis nodules were analyzed as previously described [11]. Tumors were dissected and fixed in 10% formalin and embedded in paraffin. Immunostaining was performed using antibodies for V5 (Invitrogen), p-ERK (Thr202/Tyr204; Cell Signaling Technology), β-catenin (BD Biosciences), c-Myc (9E10; Santa Cruz Biotechnology), and MMP-7 (EMD Millipore) [11]. Tissues from analysis of parental and fibulin-5-expressing H460 tumor metastasis were previously described [11].

### Statistical analysis

Statistical analyses were performed using GraphPad Prism V software. *P* values < 0.05 were considered to be statistically significant. Means + one standard deviation were displayed in the figures.

## SUPPLEMENTARY FIGURES AND TABLES



## Abbreviations

DAPI: 4′ 6-Diamidino-2-phenylindole
ECM: extracellular matrix
EMT: epithelial-mesenchymal transition
ERK: extracellular signal-regulated kinase
FAK: focal adhesion kinase
FP: first progression
GSK3β: glycogen synthase kinase 3β
GAPDH: glyceraldehyde-3-phosphate dehydrogenase
IP: immunoprecipitation
MMP: matrix metalloproteinase
NSCLC: non-small cell lung cancer
OS: overall survival
PPS: post-progression survival
RGD: Arg-Gly-Asp
RT-PCR: reverse transcriptase-polymerase chain reaction
shRNA: short hairpin RNA
siRNA: small interfering RNA
TCF-4: T-cell factor 4
TBE: T-cell factor 4 binding element
TCGA: The Cancer Genome Atlas
TIMP: tissue inhibitor of metalloproteinase
WT: wild-type
