# Supplementary material for: Fibulin-5 inhibits Wnt/β-catenin signaling in lung cancer
Source: Oncotarget. 2015 Apr 2;6(17):15022–34. doi: 10.18632/oncotarget.3609 (PMC4558133; doi:10.18632/oncotarget.3609)
Supplement: Supplementary file 1 [file oncotarget-06-15022-s001.pdf]

SUPPLEMENTARY FIGURES AND TABLES

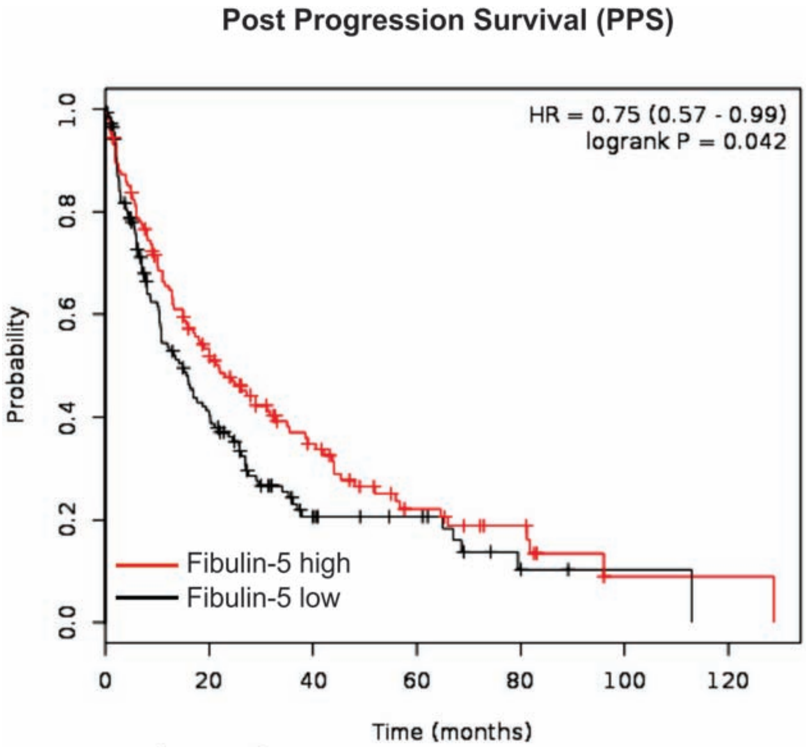

Supplementary Figure S1: Kaplan-Meier curves for comparing post progression survival (PPS) of patients with lung tumors expressing high and low levels of *fibulin-5*.

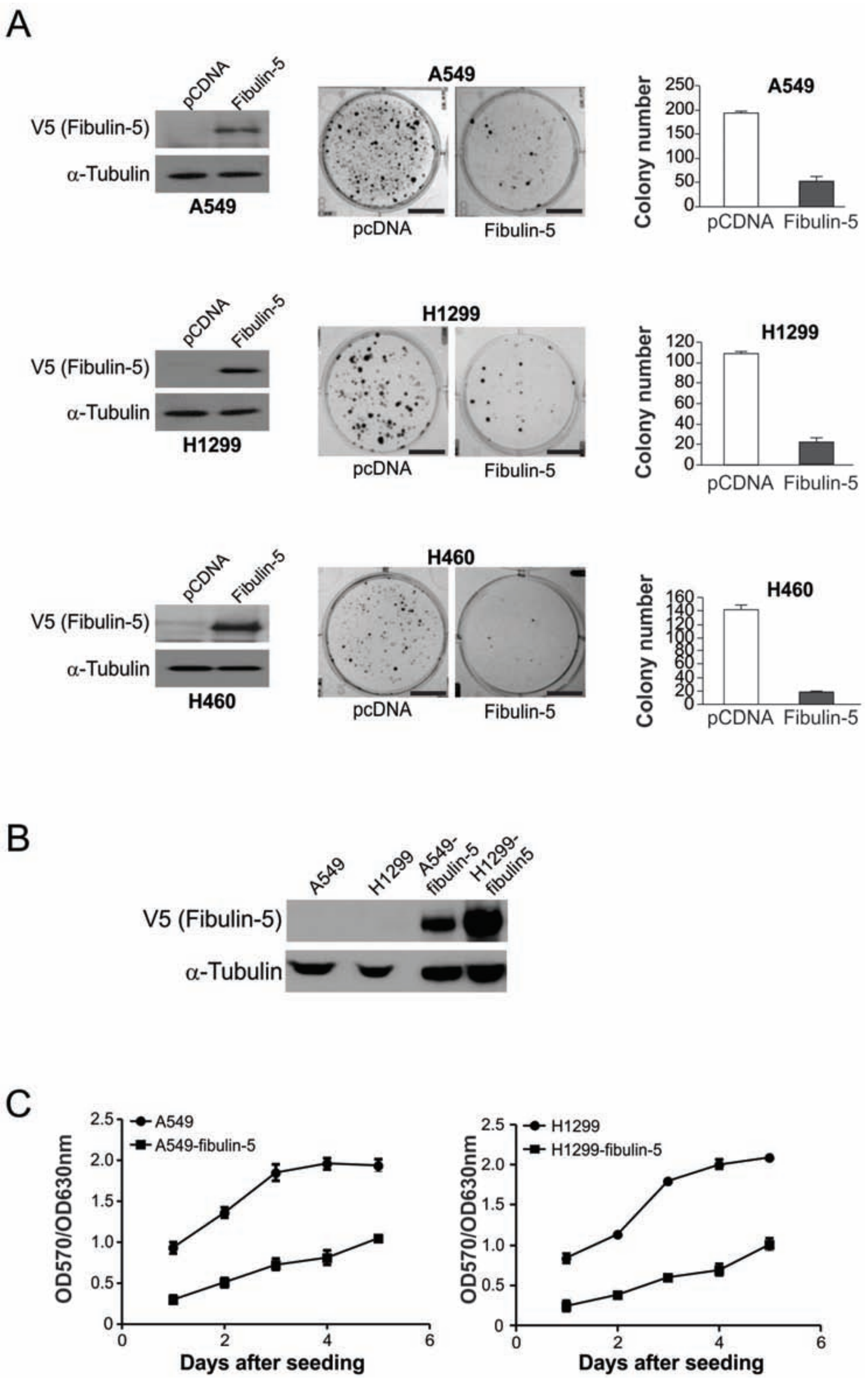

Supplementary Figure S2: Fibulin-5 inhibits lung cancer cell proliferation. A. H1299, A549, and H460 cells were transfected with V5-tagged fibulin-5 or the control pCDNA vector. *Left,* western blot analysis of fibulin-5 48 hr after transfection; *middle,* cells were plated out 48 hr after fibulin-5 transfection and selected for G418 resistance. Colonies were visualized by crystal violet staining 11 to 14 days later. Scale bars, 1 cm; *right,* quantification of colony numbers. **B.** Western blot analysis of fibulin-5 (V5) in the parental and stable fibulin-5-expressing A549 and H1299 cells. **C.** Equal numbers (3×10^3/well) of parental and stable fibulin-5-expressing A549 and H1299 cells were seeded in 96-well plates. MTT assay was used to analyze cell growth at indicated time after inoculation.

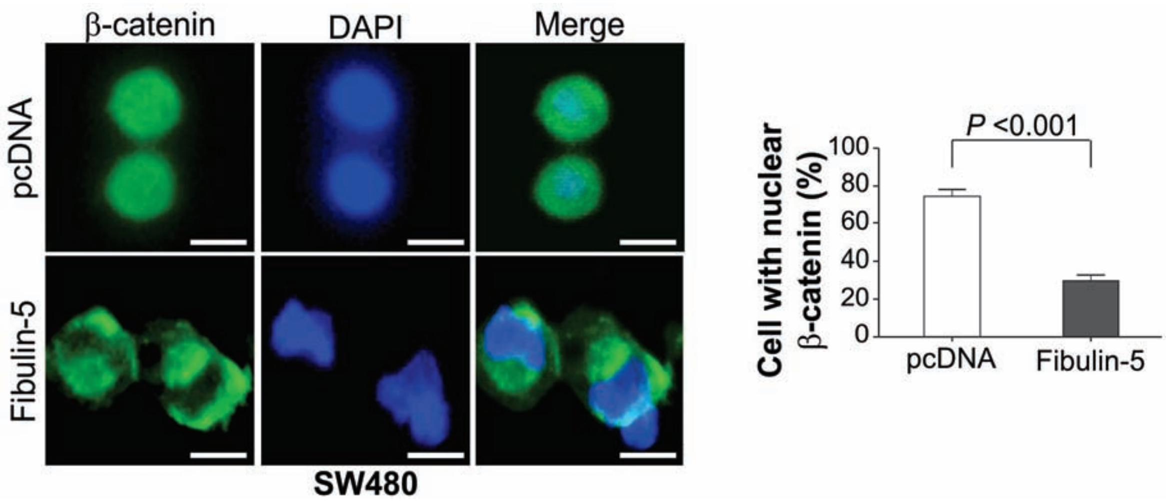

Supplementary Figure S3: Fibulin-5 inhibits β-catenin nuclear translocation. SW480 colorectal cancer cells transfected with *fibulin-5* or the control empty vector were analyzed for β-catenin by immunostaining 48 hr after transfection. *Left*, representative pictures of β-catenin immunostaining with 4' 6-Diamidino-2-phenylindole (DAPI) (blue) for nuclear counterstaining. Scale bar, 5 μm. *Right:* quantification of cells with nuclear β-catenin (P = 0.0008, Student's t test).

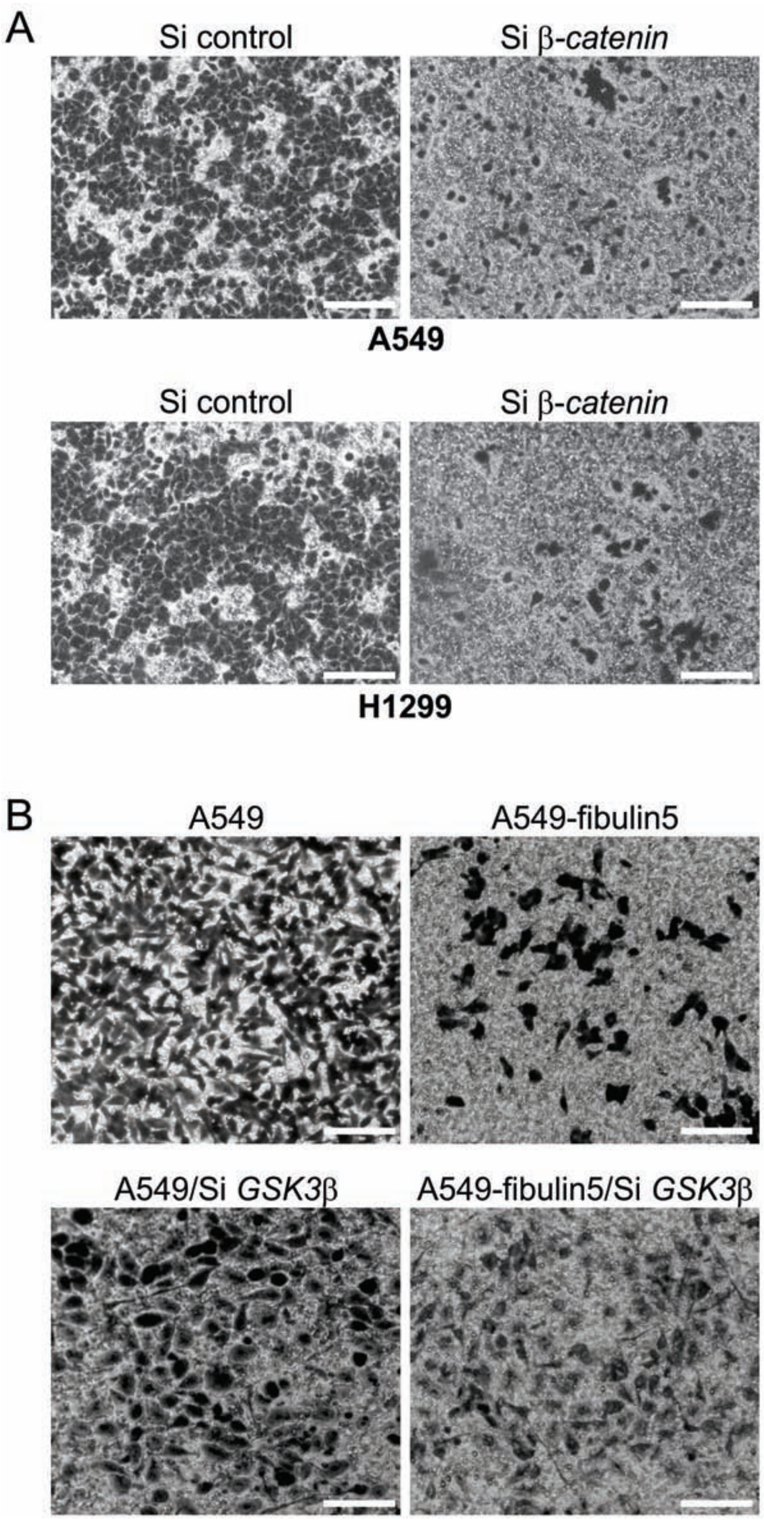

Supplementary Figure S4: The effect of fibulin-5 on lung cancer invasion is mediated by β-catenin and GSK-3β. A. Representative pictures of Matrigel invasion analysis of the parental and stable fibulin-5-expressing A549 cells with control or *β-catenin* siRNA transfection. Scale bars, 25 μm. **B.** Representative pictures of Matrigel invasion analysis of invasion of A549 and H1299 cells transfected with the control or *GSK3β* siRNA. Scale bars, 25 μm.

Supplementary Table S1. Fibulin-5 and nuclear β-catenin expression in 99 NSCLC

#	Sex	Age	Pathology	Grade	Fibulin-5 expression in tumor	β -catenin expression in tumor
1	M	46	Squamous cell carcinoma	I	−	−
2	M	46	Squamous cell carcinoma	I	+	+
3	M	50	Squamous cell carcinoma	II	−	+
4	M	60	Squamous cell carcinoma	II	−	+
5	F	64	Squamous cell carcinoma	II	−	−
6	M	75	Squamous cell carcinoma	I	−	−
7	F	66	Squamous cell carcinoma	I	−	+
8	M	62	Papillary adenocarcinoma	I	+	+
9	M	76	Mucinous adenocarcinoma	I	+	+
10	F	53	Adenocarcinoma	I	+	−
11	F	55	Mucinous adenocarcinoma	I	+	+
12	M	42	Adenocarcinoma	I	−	−
13	M	60	Papillary adenocarcinoma	I	−	−
14	F	43	Papillary adenocarcinoma	I	+	−
15	M	61	Squamous cell carcinoma	II	−	+
16	M	58	Squamous cell carcinoma	II	−	+
17	F	52	Squamous cell carcinoma	II	−	+
18	F	67	Squamous cell carcinoma	II	+	+
19	F	65	Squamous cell carcinoma	II	+	+
20	M	78	Squamous cell carcinoma	II	+	+
21	M	59	Squamous cell carcinoma	II	−	−
22	F	49	Squamous cell carcinoma	II	−	+
23	F	72	Adenocarcinoma	II	−	−
24	F	35	Adenocarcinoma	II	+	+
25	M	47	Adenocarcinoma	II	−	+
26	F	54	Adenocarcinoma	II	+	+
27	F	46	Adenocarcinoma	II	+	−
28	F	58	Adenocarcinoma	II	+	+
29	M	47	Papillary adenocarcinoma	II	−	+
30	F	63	Adenocarcinoma	II	−	+
31	F	37	Adenocarcinoma	II	−	+
32	M	47	Mucinous adenocarcinoma	II	−	−
33	M	53	Mucinous adenocarcinoma	II	−	−
34	F	55	Mucinous adenocarcinoma	II	−	−
35	M	48	Adenocarcinoma	II	−	−
36	M	56	Squamous cell carcinoma	II	+	+

(*Continued*)

#	Sex	Age	Pathology	Grade	Fibulin-5 expression in tumor	β -catenin expression in tumor
37	F	63	Adenocarcinoma	II	−	+
38	F	58	Adenocarcinoma	II	−	−
39	F	67	Adenocarcinoma	II	+	+
40	M	46	Adenocarcinoma	II	−	−
41	M	59	Papillary adenocarcinoma	II	−	−
42	M	40	Squamous cell carcinoma	II	−	−
43	F	32	Adenocarcinoma	II	−	+
44	M	40	Squamous cell carcinoma	II	−	−
45	M	57	Squamous cell carcinoma	II	+	+
46	M	72	Squamous cell carcinoma	III	+	+
47	M	58	Squamous cell carcinoma	III	−	−
48	M	53	Squamous cell carcinoma	III	+	+
49	M	41	Squamous cell carcinoma	III	−	−
50	M	54	Squamous cell carcinoma	III	−	+
51	M	50	Squamous cell carcinoma	III	+	+
52	F	49	Squamous cell carcinoma	III	−	+
53	M	51	Squamous cell carcinoma	III	+	−
54	M	65	Squamous cell carcinoma	III	−	+
55	M	72	Squamous cell carcinoma	III	−	+
56	M	51	Squamous cell carcinoma	III	−	−
57	M	53	Squamous cell carcinoma	III	+	−
58	M	56	Squamous cell carcinoma	III	−	+
59	M	56	Squamous cell carcinoma	III	+	−
60	M	61	Squamous cell carcinoma	III	−	+
61	M	66	Squamous cell carcinoma	III	−	+
62	F	45	Adenocarcinoma	I	+	+
63	M	47	Squamous cell carcinoma	III	−	−
64	M	57	Squamous cell carcinoma	III	−	+
65	M	57	Squamous cell carcinoma	III	+	−
66	M	67	Squamous cell carcinoma	II	−	+
67	F	41	Squamous cell carcinoma	III	−	+
68	F	51	Squamous cell carcinoma	II	+	+
69	F	60	Squamous cell carcinoma	III	−	−
70	M	61	Squamous cell carcinoma	III	−	−
71	M	61	Squamous cell carcinoma	III	+	−
72	M	61	Squamous cell carcinoma	III	+	−

(*Continued*)

#	Sex	Age	Pathology	Grade	Fibulin-5 expression in tumor	β -catenin expression in tumor
73	F	71	Squamous cell carcinoma	II	+	+
74	F	71	Squamous cell carcinoma	III	−	−
75	F	71	Squamous cell carcinoma	III	+	−
76	F	49	Adenocarcinoma	III	−	+
77	M	59	Adenocarcinoma	III	−	+
78	F	50	Adenocarcinoma	III	+	−
79	M	35	Adenocarcinoma	III	−	−
80	M	62	Adenocarcinoma	III	−	+
81	F	50	Squamous cell carcinoma	III	−	+
82	F	54	Squamous cell carcinoma	III	+	+
83	M	64	Squamous cell carcinoma	III	−	−
84	M	65	Squamous cell carcinoma	III	+	+
85	F	53	Squamous cell carcinoma	III	+	−
86	M	63	Squamous cell carcinoma	III	−	+
87	M	62	Squamous cell carcinoma	III	−	+
88	M	62	Squamous cell carcinoma	III	−	−
89	M	59	Adenocarcinoma	III	+	−
90	M	46	Squamous cell carcinoma	III	+	+
91	M	74	Squamous cell carcinoma	IV	−	+
92	M	60	Squamous cell carcinoma	II	−	+
93	M	54	Adenocarcinoma	III	−	−
94	M	67	Adenocarcinoma	III	−	+
95	F	56	Adenocarcinoma	III	−	−
96	F	65	Adenocarcinoma	III	+	−
97	M	51	Squamous cell carcinoma	III	−	+
98	M	45	Squamous cell carcinoma	III	−	+
99	F	39	Adenocarcinoma	III	−	+
